# State-of-the-Art Insights and Potential Applications of Cellulose-Based Hydrogels in Food Packaging: Advances towards Sustainable Trends

**DOI:** 10.3390/gels9060433

**Published:** 2023-05-24

**Authors:** Ajit Kumar Singh, Pontree Itkor, Youn Suk Lee

**Affiliations:** Department of Packaging, Yonsei University, Wonju 26393, Republic of Korea; ajitksingh.iitkgp@gmail.com (A.K.S.);

**Keywords:** biopolymer, cellulose, hydrogel, cellulose-based hydrogels (CBHs), food packaging, sustainability, biodegradability

## Abstract

Leveraging sustainable packaging resources in the circular economy framework has gained significant attention in recent years as a means of minimizing waste and mitigating the negative environmental impact of packaging materials. In line with this progression, bio-based hydrogels are being explored for their potential application in a variety of fields including food packaging. Hydrogels are three-dimensional, hydrophilic networks composed of a variety of polymeric materials linked by chemical (covalent bonds) or physical (non-covalent interactions) cross-linking. The unique hydrophilic nature of hydrogels provides a promising solution for food packaging systems, specifically in regulating moisture levels and serving as carriers for bioactive substances, which can greatly affect the shelf life of food products. In essence, the synthesis of cellulose-based hydrogels (CBHs) from cellulose and its derivatives has resulted in hydrogels with several appealing features such as flexibility, water absorption, swelling capacity, biocompatibility, biodegradability, stimuli sensitivity, and cost-effectiveness. Therefore, this review provides an overview of the most recent trends and applications of CBHs in the food packaging sector including CBH sources, processing methods, and crosslinking methods for developing hydrogels through physical, chemical, and polymerization. Finally, the recent advancements in CBHs, which are being utilized as hydrogel films, coatings, and indicators for food packaging applications, are discussed in detail. These developments have great potential in creating sustainable packaging systems.

## 1. Introduction

“Less is more”, an aphorism coined by architect Ludwig Mies van der Rohe in the 1940s, has since become a popular philosophy that applies to many aspects of life including our consumption of food and packaging. While some are essential and serve a vital purpose, others may be redundant and should be avoided. In the current scenario, food packaging waste is a major contributor to household waste, and unfortunately, much of this waste ends up in landfills. Additionally, whereas the rise of smartphones and online ordering platforms has aided the growth of the food delivery industry, it has also triggered an increase in plastic packaging waste, as many food delivery companies rely heavily on single-use plastic bags, containers, and utensils to deliver food from restaurants to the consumers’ homes [1]. Plastic packaging waste is particularly troublesome in the food packaging and delivery industry since many of these packages are designed for single use and are quite often not recycled or disposed of properly. According to Statistics Korea, the national statistical office, food delivery sales in South Korea have grown by an average of 85% over the past four years, reaching 14.3 billion dollars in sales in 2020 [1]. Since the outbreak of COVID-19, there has been a dramatic surge in demand for food delivery in Korea, culminating in even more plastic packaging waste. As a consequence of the higher demand for food delivery, approximately 2.1 billion plastic food packaging containers were produced in Korea in 2021 [2]. However, the Korean government and policymakers are making efforts to tackle the issue of plastic packaging waste in the food packaging and delivery industry. In this context, the Korean Ministry of Environment announced plans in 2020 to reduce disposable plastics use by 50% by 2030, and the government has implemented regulations aimed at promoting the use of eco-friendly packaging materials [3,4].

**Figure 1:**
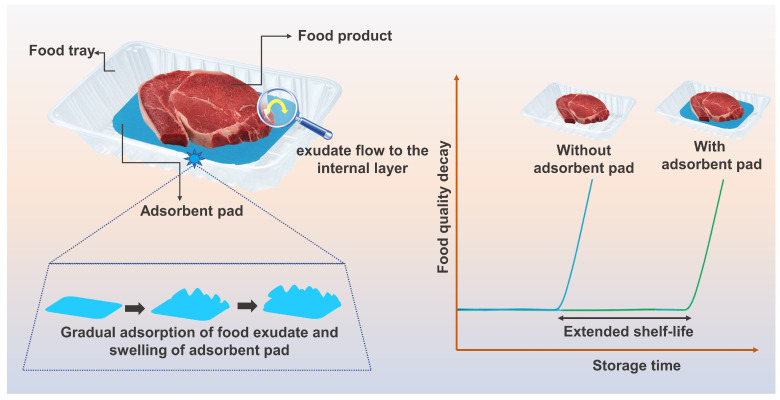
Graphical illustration of the hydrogel as an adsorbent packaging layout and their effect on food quality decay with storage time.

Bio-based polymeric hydrogels represent a promising area of research in addressing the problem of plastic waste in the food packaging segment and have become an increasingly prevalent area of research in recent years, particularly as a potential alternative to traditional petroleum-based plastics [5,6]. Unlike conventional synthetic hydrogels, which can take hundreds of years to decompose and can release harmful chemicals into the environment, bio-based polymeric hydrogels are designed to be biodegradable and environmentally friendly [7]. In general, such natural hydrogels are primarily made from proteins, lipids, and polysaccharides that come from renewable sources like plants or animals, and they are favored because of their availability, biodegradability, biocompatibility, and lack of toxicity [8,9]. These natural polymers are then chemically modified to form hydrogels, which are remarkably absorbent and can be molded into a wide range of shapes and sizes. Essentially, hydrogels are distinguished by their high water-absorption capacity and ability to retain structural integrity even when fully swollen [8]. Therefore, hydrogels are appropriate for use in applications for food packaging due to these characteristics. Controlling the humidity that results from high-water-content food is a potential application for hydrogels in food packaging. By incorporating hydrogels into the packaging material, the hydrogels can absorb excess moisture and maintain an appropriate moisture level as well as reduce the water activity (*a_w_*) of the product, which can inhibit the growth of microorganisms that cause spoilage and extend the shelf life of the food [10]. In order to preserve the freshness and shelf life of the product, Figure 1 shows a graphical illustration of this packaging and the swelling process of hydrogel-based food packaging. This illustration depicts a simulation of the exudate flow from food packages to the internal layer (hydrogel), gradual exudate adsorption in the adsorbent pad, and hydrogel swelling, taking the design of the adsorbent packaging system into account. In addition, Figure 1 demonstrates the way the application of packaging made of a hydrogel influences the rate at which food quality degrades over time. It illustrates the comparative advantages of various packaging strategies including the application of hydrogel coatings, films, or indicators. When hydrogel-based packaging is used instead of traditional packaging approaches, the graph shows a reduced rate of food quality reduction as the storage time increases. Furthermore, by integrating functional materials such as antimicrobial agents or oxygen scavengers, hydrogels can be used as active packaging [11,12,13]. These functional materials can be embedded within the hydrogel matrix and slowly released over time, assisting in the preservation of food quality and freshness.

Regarding the configuration of hydrogel matrices, a shift toward bio-based hydrogels from an extensive range of natural sources such as cellulose, starch, chitosan, and gelatin as alternatives to synthetic compounds (polyacrylamide, poly(acrylic acid), poly(sodium acrylate), and polyvinylpyrrolidone) has been explored in recent years due to the need for more sustainable and eco-friendly packaging solutions [8,14,15,16]. Among these biopolymers, cellulose and its derivatives have emerged as a popular choice for hydrogel production due to their abundance and renewability. Essentially, cellulose is a polysaccharide composed of a linear chain of β-(1→4)-linked D-glucose units that assists as the main structural component of plant cell walls and is the most abundant renewable resource on the planet. In recent years, considerable attention and research have been devoted to investigating the potential of CBHs for various applications including food packaging [12,17,18,19]. Cellulose and its derivatives are ideal materials for hydrogel production in food packaging due to its numerous advantageous properties including cost-effectiveness, moldability, water absorption, swelling ability, stimuli responsiveness, biocompatibility, and biodegradability. Furthermore, cellulose and its derivatives are versatile materials that provide substantial mechanical strength, excellent thermal stability to withstand high temperatures without degrading, and act as an ultraviolet ray protector due to their ability to absorb and scatter UV radiation [20,21,22]. However, pure cellulose-based hydrogels typically lack flexibility and functionality, limiting their wide range of applications. This is due to the fact that cellulose is a rigid and crystalline material that forms strong hydrogen bonds between the glucose units, resulting in a highly ordered and inflexible structure [23,24].

In this setting, pre-treating native cellulose with physical or chemical methods improves its solubility and reactivity, enabling the development of cellulose-based hydrogels with tailored properties and functionalities [25]. One approach is to use physical dissolution by special solvents such as ionic liquids, which can dissolve cellulose without chemical modification. Another approach is to use chemical modifications to introduce functional groups or alter the molecular structure of the cellulose, which can improve its solubility or reactivity toward cross-linking agents [26,27]. Water-soluble cellulose derivatives such as methylcellulose (MC), ethyl cellulose (EC), and carboxymethylcellulose (CMC) are commonly used to prepare cellulose-based hydrogels using physical and/or chemical cross-linking methods [20]. For example, MC and EC are cross-linked with various agents such as glutaraldehyde, genipin, or borate to form hydrogels with different properties and functionalities. CMC, on the other hand, forms hydrogels through physical cross-linking with metal ions or through chemical cross-linking with cross-linking agents such as epichlorohydrin [28,29]. Furthermore, CBHs have demonstrated great potential as intelligent packaging materials in the form of sensors, and indicators for food packaging applications due to their ability to respond to changes in the environment and functionalities in detecting as well as recording to monitor the shelf-life and quality of food products. For instance, CBHs can be embedded with indicators or sensors that change color or emit a signal when exposed to specific conditions like temperature, humidity, or gas concentration. As a result of their natural and renewable properties, high water absorption and retention capabilities, ability to reduce food waste, and versatility in design and functionality, incorporating intelligent features into cellulose-based hydrogels can help to reduce the need for traditional packaging materials and simplify the recycling process.

Notably, there is a growing interest in developing and reviewing bio-based hydrogels as food packaging materials from a sustainability point of view. This is due to increasing concerns about the negative environmental impact of traditional petroleum-based plastics, which are non-renewable, non-biodegradable, and contribute to litter and waste. Bio-based hydrogels such as CBHs offer a sustainable alternative to traditional plastics, as they are made from renewable and biodegradable sources, have lower carbon footprints, and can help to reduce food waste and improve shelf life. Additionally, the incorporation of active and intelligent features into bio-based food packaging materials is an innovative approach driven by the need to espouse sustainability, reduce waste, and improve the functionality and performance of packaging materials for next-generation smart consumers [30,31]. For example, Zafar et al. [12] recently studied carboxymethyl cellulose/gelatin hydrogel films loaded with zinc oxide nanoparticles and revealed that they were effective in antibacterial and antioxidant activities for food preservation as well as biomedical applications. Several review studies on CBHs [19,32,33,34,35] are also available. However, the majority of published reviews on CBHs refer to applications in biomedical engineering, drug delivery, tissue engineering, and wound management. While there has been a significant number of reviews on CBHs in other fields of study, there have been relatively few review studies that have focused specifically on their potential application as food packaging materials. Therefore, this review systematically explores the most recent advances and approaches of CBHs that are suitable for food packaging applications as well as the most recent trends in CBH preparation techniques and the mechanisms involved in production. In addition, the review discusses the properties and features of CBHs that make them appealing for food packaging such as their biodegradability, biocompatibility, and ability to be tailored for specific applications. Finally, the review provides an overview of the constraints and future trends in the integration of CBHs into active and intelligent packaging systems for food safety.

## 2. Hydrogel: Structural Chemistry and Classification

Hydrogels are an intriguing class of cross-linked polymeric networks with a three-dimensional structure that can retain excess amounts of aqueous solvents and biological fluids [36,37]. Recently, hydrogels have gained increasing attention from researchers across various fields of study. Over the past few years, hydrogels have become an area of focus in the food packaging industry due to their ability to enhance food quality and freshness, reduce waste, and improve food safety [38,39,40]. The increasing interest and research into hydrogels for food packaging applications highlight their potential to revolutionize the food industry and address some of the biggest challenges facing the global food supply chain. Successful examples include “absorbent pads” as an effective application of hydrogels in food packaging to regulate the moisture content in food products that have a high water content such as packaged meats, fruits, and vegetables [13,41]. The pads are placed in food packages to maintain the quality and freshness of the product by controlling the water activity and preventing the growth of bacteria and other microorganisms. While considering the application of hydrogels for active food packaging, the properties of the hydrogel such as its structure, material, and preparation methods are critical factors to consider. These factors affect the physicochemical and functional properties of the hydrogel, ultimately determining its suitability for specific applications. For example, the network structure of the hydrogel affects its mechanical strength and water absorption capacity, while the chemical composition of the hydrogel affects its antimicrobial activity and compatibility with food products [8,42,43]. Additionally, the preparation method of the hydrogel can affect its porosity, swelling behavior, and response to environmental stimuli [44]. Therefore, a comprehensive understanding of the structure, material, and preparation methods of hydrogels is crucial for designing and developing hydrogel-based active food packaging with desirable properties and functionality [45,46,47].

### 2.1. Structural Chemistry of Hydrogel

**Figure 2:**
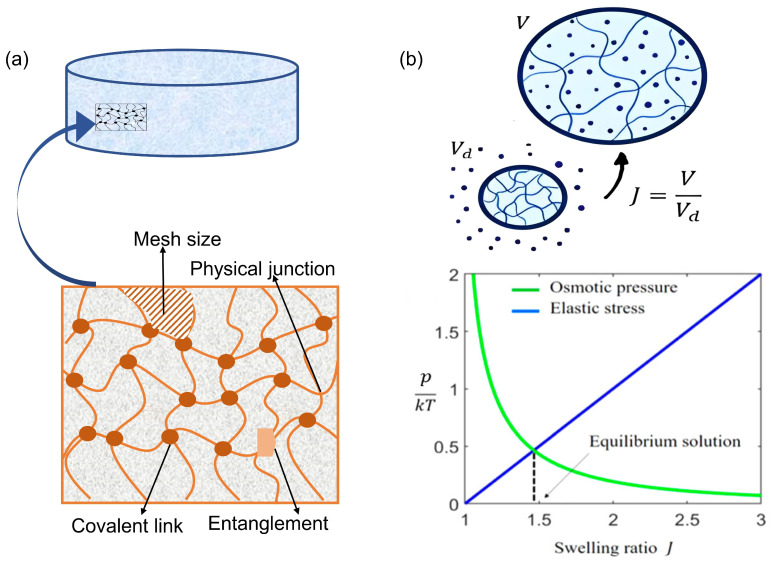
Structural chemistry of a hydrogel: (**a**) structure of a hydrogel at the molecular level; (**b**) correlation of the swelling ratio of the hydrogel with osmotic pressure and elastic stress. Adapted with permission from [56]. Copyright © 2021 American Chemical Society.

Consisting of a complex network structure of polymer chains, the hydrogel is a material that forms a porous, three-dimensional (3D) network. Such a structure is formed by the crosslinking of polymer chains of natural or synthetic polymers with the ability to absorb and retain a substantial amount of water in their interstitial frameworks [43,48]. Principally, the crosslinking process involves connecting the polymer chains with covalent or physical bonds. Covalent crosslinking involves the formation of strong chemical bonds between the polymer chains, whereas physical crosslinking involves weaker interactions such as hydrogen bonding or entanglement, as illustrated in Figure 2a. The linear distance between adjacent crosslinks is represented by the mesh size, an essential structural parameter that has a significant impact on the characteristics of the hydrogel. The mesh size determines the permeability of the hydrogel as well as its mechanical and swelling properties [49]. Furthermore, the presence of hydrophilic functional groups such as hydroxyl and carboxyl groups, accounts for the high-water absorption potential of hydrogels. In addition to hydroxyl and carboxyl groups, other hydrophilic functional groups commonly found in hydrogels include amine, ether, and sulfonate groups [34,50,51,52]. The occurrence of these functional groups also imparts advantageous features to hydrogels such as pH sensitivity or responsiveness to external stimuli such as temperature or light [53]. In addition, hydrogels are distinguished by their ability to absorb and retain large amounts of water ranging from 10 to 20% of their dry weight to several thousand times their dry weight, depending on factors such as polymer type, degree of crosslinking, and the pH and ionic strength of the surrounding environment [54,55].

The molecular weight of the polymer chains between crosslinks plays a crucial role in defining the structure of the hydrogel. When the molecular weight is high, the distance between the crosslinks increases, resulting in a larger mesh size [57,58]. This affects the ability of the hydrogel to absorb and retain water, with larger mesh sizes allowing for greater water uptake. Furthermore, the ability of water molecules to diffuse into a hydrogel network determines the degree of swelling. The amount of space available to accommodate water in the inner part of the hydrogel network determines the swelling ratio or degree of swelling (*J*), which is defined as the ratio of the volume of the swollen hydrogel (*V*) to its dry volume (*V_d_*). When a hydrogel is placed in contact with water, the water molecules diffuse into the hydrogel network, causing the polymer chains to swell and the hydrogel to expand, as shown in Figure 2b. The degree of swelling is influenced by the mesh size of the hydrogel, the molecular weight of the polymer chains, and the density of crosslinking [59,60]. For instance, a hydrogel with a high degree of crosslinking density tends to exhibit a lower swelling ratio and increased brittleness [61]. Controlling the physiochemical and mechanical properties of the hydrogel throughout the network is critical in the application of hydrogels. Such properties are influenced by the structural network of the hydrogel, which is determined by the cross-linker, comonomer, and preparation methods used. As a result, for each application, establishing the proper balance between the desired mesh size, swelling ability, and mechanical performance of the hydrogel is critical [62,63,64].

In addition to the presence of hydrophilic groups, osmotic pressure also influences the swelling behavior of hydrogels. Osmotic pressure is the pressure that develops when there is a difference in the concentration of solutes on either side of a semipermeable membrane. In the case of hydrogels, the presence of charged groups in the polymer network can create an imbalance in the concentration of ions and solutes inside and outside the hydrogel, leading to an osmotic pressure that drives water molecules into the hydrogel and causing it to swell [65,66]. As a hydrogel undergoes swelling, the polymer chains are extended by the water it absorbs, leading to the development of osmotic pressure that promotes the influx of more water into the hydrogel. However, as illustrated in Figure 2b, the polymer network also exerts an elastic restoring force that resists further swelling, creating a balance between osmotic pressure and elastic force [56]. This balance between the osmotic pressure and the elastic restoring force is often described by the Flory–Rehner theory, which relates the swelling behavior of the hydrogel to the chemical and physical properties of the polymer network including the mesh size, the degree of crosslinking, and the solubility parameters of the polymer and the solvent [67,68]. The degree of swelling of a hydrogel is influenced by a variety of factors including the chemistry and structure of the polymer network, the type and concentration of the solvent, and the presence of any additives or functional groups that can interact with water [69,70]. The degree of swelling of a hydrogel has a significant impact on its performance in food packaging applications. For example, the degree of swelling affects the rate and extent of active components released from the hydrogel. In addition, the degree of swelling influences the mechanical properties and biocompatibility of the hydrogel scaffold [71,72,73]. Therefore, understanding and controlling the degree of swelling is an important consideration in the design and optimization of hydrogels for food packaging applications.

### 2.2. Classification of Hydrogel

**Figure 3:**
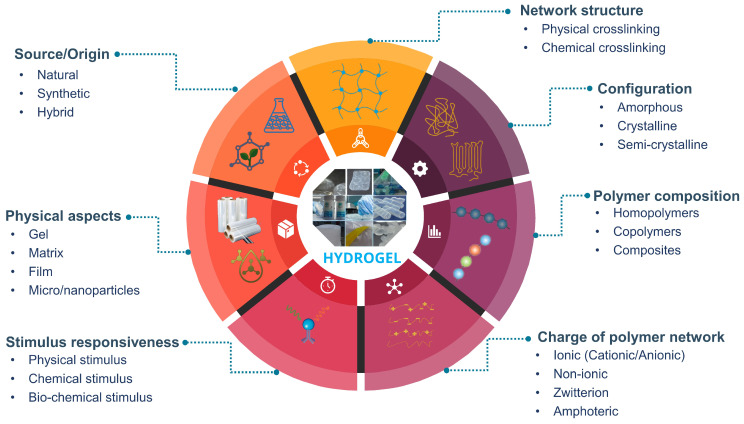
Schematic representation of the classification of hydrogels.

Although hydrogel classification can be complex and multifaceted, as different criteria may be relevant for different applications, a systematic understanding of hydrogel classification is vital since it offers a framework for understanding and selecting hydrogels with specific properties and applications. Hydrogel classification, for example, is critical for optimizing hydrogel performance in packaging applications by selecting or designing hydrogels with application-specific properties such as high-water absorption capacity, biocompatibility, and the ability to form a barrier against gases and vapors. Various classification criteria can be employed to discern the difference between hydrogels. These can be categorized as renewable or non-renewable based on their sources, which differs from a classification system that depends on their origin (natural and synthetic). Other hydrogel classifications include network structure, configuration, polymer composition, polymer network charge, stimulus responsiveness, and physical aspects [14,36,74], which are depicted schematically in Figure 3.

Based on their source or origin, they are classified as natural or synthetic polymers, or an amalgamation of both, in which case they are referred to as hybrid, which is a significant consideration in terms of the selection and design of hydrogels. Currently, most hydrogel matrices are derived from synthetic sources that originate from petroleum derivatives such as polyacrylamide, poly(sodium acrylate), poly(acrylic acid), and polyvinylpyrrolidone [8,75]. These synthetic hydrogels exhibit exceptional mechanical and chemical properties that make them appropriate for a broad range of applications. On the other hand, hydrogels derived from natural polymers such as cellulose, collagen, gelatin, alginate, and chitosan are appealing due to their superior biocompatibility, biodegradability, and lower toxicity [76,77]. Nevertheless, these hydrogels tend to have inferior mechanical properties and stability. Therefore, researchers are focusing on enhancing the properties of natural hydrogels or creating hybrid hydrogels that combine the advantageous features of both natural and synthetic hydrogels [78,79].

While hydrogels can be classified in a variety of ways, they are primarily classified based on network structure as constructed by cross-linking networks; thus, they are categorized into two groups depending on cross-linking: (i) physically cross-linked or self-assembled hydrogels and (ii) chemically cross-linked hydrogels. Physically cross-linked hydrogels, also known as reversible or self-assembled hydrogels, have gained popularity in recent years due to several advantages over chemically cross-linked hydrogels. One of the key benefits is their relatively simple and cost-effective manufacturing process, which does not require the use of cross-linking agents or other chemical reactions [36]. Physically cross-linked hydrogels are formed through reversible, non-covalent interactions such as hydrogen bonding, van der Waals forces, or physical entanglements between the polymer chains. These interactions allow the hydrogel to form a three-dimensional network structure that can absorb and retain large amounts of water [80]. Chemically cross-linked hydrogels, on the other hand, are formed via covalent bonds between different polymer chains, resulting in an irreversible and stable network structure. Covalent bonds are formed through a variety of chemical reactions including free radical polymerization, Michael addition, and click chemistry, which can be tailored to create hydrogels with specific properties such as mechanical strength, porosity, or biodegradability [81,82]. Cross-linking agents such as glutaraldehyde, epichlorohydrin, adipic acid dihydrazide, and polyaldehydes, among others, are commonly used in the synthesis of chemically cross-linked hydrogels [83]. These cross-linkers react with functional groups on the polymer chains such as amino or carboxyl groups to form covalent bonds that link the polymer chains together and form the hydrogel network [83,84].

Furthermore, depending on the configuration or physical structure, hydrogels are classified in terms of crystallinity or amorphousness, which refers to the arrangement of the polymer chains within the hydrogel matrix. Crystalline hydrogels have a highly ordered and structured arrangement of polymer chains, similar to that of a crystal. This results in high mechanical strength and stiffness, but also reduces the swelling capacity and flexibility of the hydrogel [85,86]. Amorphous hydrogels, on the other hand, lack long-range order and have a more disordered arrangement of polymer chains. This leads to increased flexibility and swelling capacity but reduced mechanical strength [87,88]. The physical structure of a hydrogel has a significant impact on its properties and potential applications. Highly crystalline hydrogels, for example, may be suitable for load-bearing applications, whereas more amorphous hydrogels may be better suited for active and smart packaging applications requiring flexibility and swelling capacity. Hydrogels can also be classified based on their polymeric composition, which refers to the type of polymer used to create the hydrogel network. According to polymeric composition, hydrogels can be categorized based on homopolymer (the polymer network derives from a single species of monomer), copolymers (comprising two or more different monomer types, arranged along the polymer network chain), and composite or multipolymer interpenetrating polymeric hydrogels (IPN) [83,89]. As shown in Figure 3, hydrogels are further classified based on several factors including the charge of their polymer network (such as ionic, non-ionic, zwitterionic, or amphoteric), their responsiveness to different stimuli (such as physical, chemical, or biochemical), and their physical properties (such as being in the form of a gel, matrix, film, or micro/nanoparticles), as shown in Figure 3. These classification systems are critical for guiding the selection and design of hydrogels depending on the desired application and properties.

## 3. Sustainable Hydrogels from Cellulose: Synthesis Routes and Characterization

Excessive use of petroleum-based and non-biodegradable materials has resulted in a myriad of environmental issues such as pollution, global warming, and resource depletion. As a result, there is an urgent need to investigate and promote alternative materials that are both sustainable and environmentally safe [90]. Biopolymers have emerged as a promising approach to address these challenges. Biopolymers are derived from renewable sources such as plants, animals, and microorganisms, and are biodegradable. They offer several advantages over synthetic polymers such as reduced environmental impact, enhanced biocompatibility, and improved sustainability [91,92]. Among the various biopolymers, cellulose is one of the most abundant and versatile materials in nature. In terms of food packaging materials, cellulose, and its derivatives have excellent properties such as hydrophilicity, mechanical strength, biodegradability, low toxicity, and an abundance of hydrophilic functional groups (such as hydroxyl, carboxyl, and aldehyde groups), making them a promising material for the synthesis of biocompatible hydrogels [18,93,94]. Moreover, the widespread availability and low cost of cellulose and its derivatives make them a viable alternative to petroleum-based plastics, which are non-renewable and have negative environmental consequences. However, there are still some challenges associated with CBHs for packaging applications. One of the major challenges is to develop hydrogels that have sufficient mechanical strength to withstand handling and transport while maintaining their barrier properties [39]. Another challenge is to ensure that the release of antimicrobial agents, antioxidants, and other additives from CBHs is controlled in order to improve food preservation and shelf life [12,95,96]. As a result, the first and most important step toward effective barrier properties and controlled release ability is to synthesize CBHs with sufficient mechanical strength and handling ability to release antimicrobial agents, antioxidants, and other additives, making them suitable for various types of foods.

### 3.1. Synthesis of Cellulose-Based Hydrogels (CBHs)

Cellulose-based hydrogels (CBHs) are synthesized from various sources including cellulose and its derivatives such as methyl cellulose (MC), hydroxypropyl cellulose (HPC), hydroxypropyl methyl cellulose (HPMC), and carboxymethyl cellulose (CMC). These derivatives have modified chemical structures that can improve the solubility, viscosity, and other properties of cellulose, making them useful in a range of applications [97,98]. Furthermore, ester derivatives of cellulose such as acetate trimellitate, acetate phthalate, hydroxypropyl methyl phthalate, hydroxypropyl methyl phthalate acetate succinate, etc., can also be used to create CBHs [43,99]. These derivatives have different properties and can be used to tailor the properties of CBHs for specific applications. CBHs are also synthesized by combining cellulose with other polymers such as polyelectrolyte complexes, or blending with other polymers [100,101]. This provides the potential to improve the mechanical and other properties of the resulting CBHs. One of the challenges of synthesizing hydrogels from cellulose or its derivatives is that they are not easily soluble in common solvents, particularly native/pure cellulose [43,97]. This makes dissolving and processing these materials into hydrogels more difficult. To address this challenge, researchers have developed various methods for dissolving cellulose or its derivatives. Some of the most commonly used solvents include alkali/urea or thiourea, LiCl/dimethylacetamide, N-methylmorpholine-N-oxide, and ionic liquids. Alkali/urea or thiourea solvents function by disrupting the hydrogen bonds between the cellulose molecules, whereas LiCl/dimethylacetamide can dissolve both cellulose and some of its derivatives [102,103]. N-methylmorpholine-N-oxide is another solvent that has been used to dissolve cellulose, and ionic liquids are a relatively new class of solvents that have been shown to dissolve cellulose and its derivatives [103,104].

The process of synthesizing CBHs has evolved over the years, with different approaches being employed depending on the specific crosslinking and processing techniques used. This has been conducted in order to achieve the desired properties and applications of the hydrogel, which can vary depending on the intended use of the material. Principally, there are various approaches including physical and chemical crosslinking, and Table 1 depicts the synthesis methods, crosslinking mechanisms, and characteristics observed for different types of cellulose used in the fabrication of CBHs for various applications. These methods can improve the viscoelasticity and mechanical properties of hydrogels, altering the properties of the hydrogel to match specific criteria for packaging applications. Physical crosslinking involves the formation of CBHs through non-covalent interactions such as hydrogen bonding, van der Waals forces, or the physical entanglement of polymer chains [36,97]. This method has gained increasing interest in recent years due to the wide range of properties and applications it offers compared to chemically crosslinked polymers. In addition, physical crosslinking does not require the use of any chemical reagents, making it a simple and cost-effective method for producing hydrogels. Such crosslinking can be employed through different methods including heating or cooling a polymer solution, complex coacervation, freeze–thawing, hydrogen bonding, ionic interaction, or self-assembly. One example of physical crosslinking is the synthesis of CBHs using freeze–thaw cycles [105,106]. In this method, cellulose is dissolved in a suitable solvent, and the resulting solution is subjected to repeated cycles of freezing and thawing. This process results in the formation of a hydrogel due to the aggregation of cellulose chains. In one study, a high-performance CMC-based hydrogel film with desirable stretchability, UV-blocking, antioxidant, self-healing, and adhesion features was developed using the freeze–thaw method and was found to be effective for food packaging [17].

Although physically crosslinked hydrogels have the advantage of not requiring crosslinking agents or chemical modification, they have limitations, particularly in terms of their mechanical strength, which is crucial for many properties [119,120]. In contrast, chemically crosslinked hydrogels are robust, impede dilution and diffusion, and have higher stability because they cannot dissolve in water without breaking covalent bonds [8,21]. Chemical crosslinking involves the use of chemical reactions to form covalent bonds between polymer chains within the hydrogel network. In this context, chemical crosslinking can be achieved through various methods including grafting, the use of crosslinking agents such as citric acid, epichlorohydrin, and glutaraldehyde, enzyme crosslinking, radical polymerization, and radiation crosslinking [21,121]. To synthesize CBHs, suitable chemical modification techniques are employed, which depend on the crosslinking agent used and reaction variables like temperature, pH, and time. Through covalent crosslinking of the cellulose polymer chains, these approaches are used to create specific hydrogel properties such as swelling behavior, mechanical strength, and degradation rate. The crosslinking agents react with the functional groups on the polymer chains such as the hydroxyl groups on cellulose to form covalent bonds, resulting in a three-dimensional network structure. An example of the use of chemical crosslinking in CBHs is the synthesis of hydrogel films based on hydroxyethyl cellulose (HEC) and zinc oxide (ZnO), which employed citric acid as a crosslinking agent. These hydrogels revealed excellent swelling and hydrophilicity as well as potential antibacterial characteristics, making them appropriate for application as food packaging materials [112].

Furthermore, grafting is another promising approach in the development of CBHs for food packaging applications [19,122]. Principally, the grafting method for CBHs entails the covalent attachment of polymer chains such as polyethylene glycol (PEG) or poly (acrylic acid) (PAA) to the surface of the hydrogel network [29,123]. This covalent attachment is achieved through various techniques such as chemical grafting, radiation-induced grafting, or enzymatic grafting. Grafting allows for the modification of the surface properties of the hydrogel, improving interactions with food packaging and enabling the controlled release of bioactive molecules in active packaging applications. Additionally, grafting improves the mechanical properties of the hydrogel, making it more suitable for food packaging applications that require specific mechanical properties [124,125]. Moreover, grafting is used to introduce functional groups into the hydrogel, allowing for the development of smart hydrogels that respond to external stimuli such as pH or temperature, which can be beneficial for food packaging applications where environmental conditions need to be controlled to maintain food quality and safety [126]. Other chemical crosslinking techniques employed in the development of CBHs, as described in Table 1, include radiation crosslinking and radical polymerization [115,116,117,118]. Radiation crosslinking involves exposing the cellulose-based hydrogel to ionizing radiation such as gamma rays, X-rays, or electron beams, which creates free radicals that react and form crosslinks between the polymer chains [127]. The advantage of radiation crosslinking is that it does not require any chemical crosslinking agents, and it can be used to crosslink hydrogels in a controlled and homogeneous manner. While radiation crosslinking is a useful technique for synthesizing CBHs, it does have limitations such as requiring specialized equipment and facilities, potential damage to polymer chains, is a time-consuming process, and high cost [21,128]. Radical polymerization, on the other hand, involves the use of chemical initiators that generate free radicals, which then react with the monomers to form polymer chains that crosslink with the cellulose-based hydrogel network [129,130]. The advantage of radical polymerization is that it allows for a high degree of control over the crosslinking process, allowing for the precise tuning of the properties of the resulting hydrogel [131].

In general, the selection of a crosslinking technique for the synthesis of CBHs is determined by the specific application and the desired properties of the hydrogel. Although physically crosslinked hydrogels do not require crosslinking agents or chemical modification, they need further modifications to improve high mechanical strength. Alternatively, while chemical crosslinking is an efficient approach for synthesizing CBHs with better mechanical and thermal properties, its toxicity and lack of biodegradability make it undesirable for use in food packaging applications. Instead, researchers are more interested in investigating alternate methods for creating hydrogels that are safer, more sustainable, and more suited for packaging applications through the integration of cellulose and its derivatives with novel synthesis techniques.

### 3.2. Characterization of CBHs

The utilization of bio-based hydrogels in food packaging in various forms such as coatings, films, and composites offers a promising solution to the environmental issues related to the usage of non-renewable and non-degradable petroleum-based polymers [132]. However, when utilizing such hydrogels for food packaging, it is crucial to consider their functional properties for the intended application. In this context, the fundamental characteristics of the hydrogel such as its mechanical strength, high absorption or superabsorbent capacity, wettability, and barrier properties are crucial factors to consider alongside cost-effectiveness, sustainability, and biodegradability, regardless of whether CBHs are utilized for traditional, active, or smart packaging purposes [8,21]. In general, numerous factors such as the cellulose source, crosslinking method, crosslinking density, degree of substitution, and processing conditions, influence the performance of CBHs for food packaging applications [133]. The hydrogel characteristics including high water absorption capacity, biocompatibility, biodegradability, mechanical strength, and functional properties can be tailored to meet the needs of various food packaging applications by identifying and optimizing these factors. One of the key features of CBHs is the swelling behavior, which is an important characteristic that determines its water uptake capacity and retention ability. CBHs have exhibited tremendous potential in absorbing large amounts of water, up to 1000 times of their original dry weight, making them ideal for use in food packaging applications where moisture control is crucial [43,71]. For instance, a CMC-based hydrogel film prepared with polyvinylpyrrolidone (PVP) as a binding agent demonstrated significant water retention as well as water uptake capacity during a swelling and deswelling study and was referred to as a promising food packaging material for fruits and vegetables to keep them fresh for longer periods. In addition, the presence of PVP in the CMC-based hydrogel improved the mechanical characteristics during the hydrothermal stage in the various testing temperature and relative humidity conditions [134]. Furthermore, the swelling behavior of CBHs is controlled by varying the crosslinking density, degree of substitution, and type of cellulose used. Crosslinking density affects the degree of swelling, with a higher crosslinking density resulting in a lower degree of swelling [19]. Wettability, or the ability to interact with water or other fluids, is another crucial property affecting the features of CBHs for food packaging applications. For applications requiring fluid absorption or adhesion, high-wettability is preferred for hydrogels, whereas hydrophobicity or repellency is preferred for low-wettability applications [135]. Table 2 summarizes some of the key properties and significance of CBHs that are important for food packaging applications.

In terms of the mechanical strength of CBHs, the endurance and performance of packaging materials is a crucial characteristic as it determines the ability of the material to withstand external forces and maintain its structural integrity during handling, transportation, and storage [19,21]. For instance, in food packaging applications where different food types have variable moisture levels, the CBHs must be able to maintain their integrity and perform their intended function under a range of temperatures and humidity levels. In this context, a highly elastic and versatile hydrogel film comprised of CMC, polyvinyl alcohol (PVA), poly(ethylene imine) (PEI), and tannic acid (TA) for food packaging applications possessed a remarkable tensile strain of up to 400% without rupture [17]. Furthermore, several studies have revealed that a range of factors including the degree of polymerization of the cellulose chains, type, and concentration of the crosslinking agent, choice of solvent, and fillers as well as the method of preparation and processing have a substantial effect on the mechanical strength of CBHs [143]. These factors have a vital influence in determining the mechanical properties of CBHs, and identifying their effects facilitates the development of hydrogels with improved mechanical strength. Similarly, the thermal stability of CBHs is critical for developing food packaging materials that can endure high temperatures during processing, storage, and transportation without compromising the quality and safety of the food product. Different analytical techniques including differential scanning calorimetry (DSC), dynamic mechanical analysis (DMA), and thermogravimetric analysis (TGA), are used to analyze this. These methods offer important insights into the thermal behavior of CBHs including their glass transition temperature, thermal degradation temperature, and storage modulus as a function of temperature. One study that investigated the synthesis of a CMC-based hydrogel packaging film used TGA and DSC analyses to establish how thermal stability improved when tannic acid was incorporated. This investigation additionally confirmed that CMC and tannic acid interacted effectively together to produce the hydrogel [17]. In addition, structural features as well as the chemical compositions, molecular configurations, and functional groups of the developed CBHs can be examined as well as identified using physical, morphological, and chemical characterization approaches [139,140,141,142]. Such properties offer significant insights into interpreting the characteristics of CBHs and enhancing their functionality as food packaging materials.

## 4. Application of CBHs in Food Packaging System

Packaging is an integral component of the food supply chain in protecting and preserving food products from contamination, spoilage, and damage during transportation and storage. However, inadequate, or inappropriate packaging results in significant food waste and loss, affecting not only the quality of food products but also the environment and the economy. According to estimates by the United Nations Food and Agriculture Organization (FAO), approximately one-third of all food produced in the world is lost or wasted every year, amounting to around 1.3 billion tons of food. Of this, around 40% of food waste occurs in the United States alone, with packaging being a major contributor [144,145]. In recent years, there has been a surge of interest in investigating the potential applications of hydrogels in food packaging. Although hydrogels have been utilized since the 1960s in a variety of industries, the usage of hydrogels in food packaging is a comparatively recent advancement. One of the earliest reported applications of hydrogels in food packaging emerged in the late 1990s when researchers began investigating the usage of superabsorbent hydrogels in meat packaging [146]. The rationale was to incorporate these hydrogels in the packaging to absorb excess moisture, extending the shelf life of meat and minimizing the risk of spoilage. This was deemed as a promising application of hydrogels in food packaging, which prompted the prospect for further investigations into their potential.

**Figure 4:**
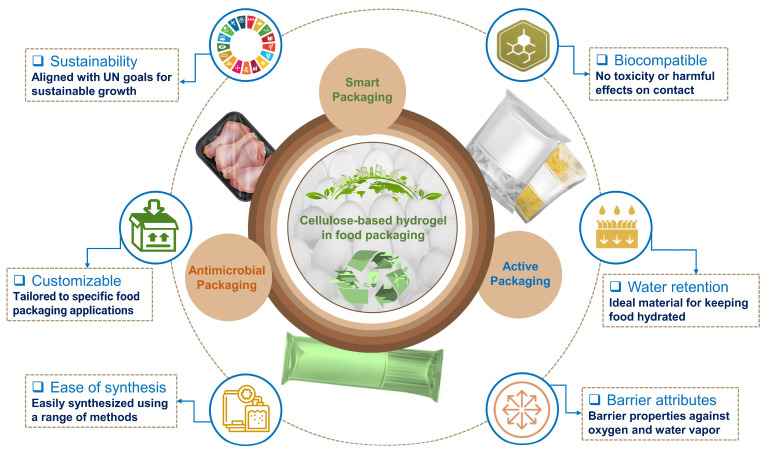
Graphical depiction of the characteristics and application of CBHs in sustainable food packaging systems.

Currently, synthetic materials are used to create the majority of hydrogels, with petroleum derivatives like polyacrylamide, poly(sodium acrylate), poly(acrylic acid), polyethylene glycol, and polyvinylpyrrolidone being the most frequently used [14,29]. These synthetic compounds offer several advantages over natural polymers including better control over their properties and easier scalability for large-scale production. Despite their many advantages, synthetic hydrogels do have some drawbacks. Their potential impact on the environment is one of the primary concerns, as many synthetic materials are not biodegradable and last a long time in the environment [121,147]. This has led to an increasing trend in developing bio-based hydrogels using sustainable and natural resources. Several natural polymers have been explored for bio-based hydrogel production including cellulose, starch, chitosan, and gelatin. These polymers offer several advantages over synthetic compounds including reduced environmental impact and improved biocompatibility. In comparison to other natural polymers, cellulose is often preferred among other natural polymers for the synthesis of CBHs due to their abundance, renewable nature, biocompatibility, high water absorption capacity, good mechanical properties, gas and vapor barrier properties, and biodegradability [97,148]. These properties make them sustainable and eco-friendly materials for food packaging applications, which can aid in reducing food waste and promoting a circular economy. Figure 4 illustrates the adoption of CBHs due to their distinctive characteristics in food packaging to improve shelf life and food safety, with an emphasis on modern applications including active and smart packaging. While CBHs provide numerous merits for use in food packaging, some limitations need to be considered during the development of cellulose-based packaging hydrogels. Their sensitivity to environmental variables like temperature and humidity, which can alter their stability and mechanical characteristics, is one of the key concerns [43,149]. However, researchers are investigating distinctive approaches to enhance the performance of cellulose-based hydrogel structures that can be used for a variety of packaging applications through the use of supporting biopolymers, novel crosslinking agents, and techniques.

The application of CBHs in food packaging has recently become increasingly popular as a result of evolving market trends and customer demands for products that are safe, healthy, and of a high standard. This trend has evolved continuously as innovative packaging approaches have been devised. In this regard, numerous investigations have been conducted involving the application of CBHs developed from cellulose for extending the shelf life and to enhance the food product quality. Table 3 provides an overview of recent applications of CBHs for preserving various foods as a sustainable and effective approach to food packaging systems. For instance, a remarkably stretchable and versatile CMC-based hydrogel film combined with polyvinyl alcohol (PVA), poly(ethylene imine) (PEI), and tannic acid (TA) was demonstrated to be beneficial in extending the shelf life of fresh fruits than traditional food packaging films, which have a limited ability to stretch and a relatively simple functionality [17]. This study reported that the developed hydrogel film could withstand an exceptional 400% tensile strain without rupturing, which is substantially higher than that of typical food packaging films. The optimized hydrogel film also delivered several additional advantages such as self-healing, UV blocking, excellent adhesion strength, and antioxidation properties as well as good water and oxygen barrier features. When stored under ambient conditions, these features helped to extend the shelf life of fresh strawberries, mangoes, and cherries by at least one week. Another study indicated that fabricating hydrogel films based on bacterial cellulose (BC) for food packaging applications improved the preservation of blueberries for 15 days [150]. This study, in an interesting move, focused on combining biopolymers to create three-dimensional CBH structures for the enhancement of mechanical and barrier properties. As a result, guar gum (GG), CMC, and polyvinyl pyrrolidone (PVP) were all included to enhance the features, which led to an improvement in barrier property as well as elastic and load-bearing capacity.

Active packaging that combines nanoparticles (ZnO, TiO_2_, and silica) into composite films to improve thermomechanical, antioxidant, and antibacterial properties has been the subject of substantial research in recent years [156,157]. In particular, enhancing the quality and shelf life of packaged food products has been the theme of several studies. Concerning the antibacterial and antioxidant properties of zinc oxide nanoparticles (ZnO-NPs), CMC and gelatin-based hydrogel films loaded with ZnO-NPs demonstrated strong antioxidant activity and antibacterial properties against Escherichia coli and Staphylococcus aureus, making them suitable for active food packaging applications [12]. In this investigation, the moisture content, water vapor permeability, mechanical strength, and thermal stability of CBHs were all improved through the inclusion of ZnO-NPs. Furthermore, ethylene gas is released during the packaging of fruits and vegetables like apples, mangoes, tomatoes, and bananas, which reduces the shelf life of the produce and accelerates ripening [158]. Another essential component, humidity, serves a key role in the preservation and shelf life of many packaged foods, particularly garden fruits. Taking this into consideration, hydrogel sorbents based on CMC were developed utilizing nanofiber cellulose and potassium permanganate. These materials reduced the humidity inside the packaging while extending the shelf life of bananas by absorbing the ethylene generated during storage [151]. Another notable application of hydrogels in food packaging is in coatings, which comprise the direct formation of a network arrangement as a surface coating on a paper-based substrate as an eco-friendly and simple method of producing functional paper. In one study, TOCNF and nisin were used to create a hydrogel coating that had strong antibacterial qualities and prohibited *Listeria monocytogenes* from growing on the external layer of the cheese [153].

Hydrogels have also found a prominent role in intelligent food packaging, where it can be utilized as a tool in the form of smart indicators to monitor changes in the condition of packed food products such as freshness, spoilage, and ripeness as well as microbial activity and transport and storage history [159]. By examining the metabolites created in fresh food by microbial growth such as organic acids, CO_2_, volatile basic nitrogen, or sulfur compounds, hydrogel indicators can identify food spoilage. Due to their beneficial characteristics including biocompatibility, biodegradability, ease of processing, high water-retention property, and good mechanical property, CBHs are a good substrate and matrix in the preparation of hydrogel indicators. These indicators are prepared by incorporating stimuli-responsive functional materials into the cellulose-based hydrogel substrate or dispersing them throughout the hydrogel matrix [19,21]. The quality of the food product inside the package is subsequently displayed by a visible color change in these indicators. The hydrogel used in such indicators is typically designed to swell or shrink in response to changes in the environment, which triggers a visible color change or other signals. For instance, a colorimetric indicator for identifying the deterioration of minced pork was developed using CBHs consisting of methylcellulose, alginate, and bromothymol blue. The hydrogel indicator showed total volatile basic nitrogen (TVB-N) accumulation in the package on the sixth day of minced pork preservation at 4 °C as the color changed from orange to yellow [154]. In another investigation on hydrogel indicators, a hydrogel made of sugarcane bagasse nanocellulose was developed as a colorimetric freshness indicator for keeping track of chicken breast deterioration [38]. The developed hydrogel indicator carried bromothymol blue/methyl red pH-responsive dyes, which switched color depending on the freshness of the chicken specimen. The study correlated chicken degradation with rising CO_2_ levels in the tightly packed case.

While CBHs films and indicators have shown promising potential in food packaging applications, there are also some challenges that need to be addressed. One challenge is the development of cost-effective and scalable production methods for CBH films and indicators [160]. Current methods are often time-consuming and require specialized equipment, which can limit their practical applications in the food industry. Another challenge is the optimization of the properties of CBH films and indicators such as their sensitivity and selectivity to specific metabolites [161,162]. Improvements in these properties can enhance their accuracy and reliability in detecting food spoilage. Moreover, there is a need for more comprehensive studies on the safety and toxicity of CBH films and indicators when in contact with food. This is particularly vital with the increasing demand for sustainable and eco-friendly packaging materials.

## 5. Conclusions and Future Outlook

Over the past few years, the need for fresh, wholesome, and secure food products has spurred the emergence of novel packaging systems that employ bio-based hydrogels with active and intelligent properties. The goal of these systems is to prolong shelf life while simultaneously providing up-to-date information about the contents of packaged foods. This progress represents an encouraging stride for the food industry, and it is anticipated that further innovations in this field will continue to surface in the coming years. Moreover, these cutting-edge packaging approaches aid in ensuring food safety and traceability as well as promoting sustainability by reducing food waste, minimizing the environmental impact of packaging, and leading to more sustainable food chains. Cellulose is the most prevalent natural biopolymer on the planet, which makes it an excellent choice as a sustainable and renewable raw material for many applications including hydrogel synthesis for food packaging. Among the other bio-based polymers, cellulose has numerous benefits including good processability, high water retention capacity, biocompatibility, biodegradability, and high mechanical strength. Such characteristics make cellulose an appealing biopolymer for the synthesis of hydrogels. CBHs are produced by crosslinking cellulose or its derivatives with a crosslinker to form a three-dimensional network structure that can absorb and hold excess amounts of water or fluids. In this review, the most recent advancements in the synthesis, characterization, and uses of CBHs for food packaging applications are summarized. The classification, structural chemistry, and distinctive features of hydrogels are briefly discussed in order to emphasize the significance of the widespread adoption of hydrogels including the food packaging field.

Currently, there is influential and substantial progress in the field of research on CBH films, coatings, and indicators for food packaging applications. Numerous investigations have reported the successful fabrication and evaluation of CBH films and coatings with better mechanical and barrier properties as well as improved water retention capacity. These films and coatings have been demonstrated to effectively prolong the storage durations of a variety of food items such as fruits, vegetables, and meats by functioning as a barrier against microbial growth, moisture, and oxygen. In addition, CBH indicators have also been developed to track the freshness and quality of packaged foods. These indicators are capable of noticing alterations in the condition of food products such as spoilage, maturity, and microbial activity, and they exhibit a visual color shift to convey information about the quality of foods. Several studies have demonstrated that CBH indicators have the scope to be applied appropriately in a variety of food packaging settings.

Despite the numerous positive aspects of CBHs, there are still a few obstacles that need to be sorted out to promote their continued adoption in applications for food packaging. For example, the mechanical and barrier-related attributes of CBH films and coatings still need to be improved to accommodate the specific requirements of various food products. Another major challenge is the cost of production. CBHs can be more expensive to produce than traditional plastic-based packaging materials, which can make them less attractive to manufacturers. Furthermore, the source of cellulose and the processing conditions can have a substantial influence on the characteristics of CBHs, consequently making it difficult to ensure conformity in both cost of production and performance.

In conclusion, while there are still some challenges that need to be addressed to ensure their widespread adoption, the benefits of using CBHs for food packaging are clear and offer a promising pathway for creating more sustainable and environmentally friendly packaging solutions. With so much scope for development in the future, CBHs have the potential to take the lead in supporting the creation of sustainable food packaging systems.

## Figures and Tables

**Table 1:** Various methods for the development of cellulose-based hydrogels.

Synthesis Routes	Crosslinking Mechanism	Types of Cellulose	Characteristics	References
Physical crosslinking	Freeze thawing	Native cellulose	One-pot supramolecular bio-based hydrogels with high strength and pH sensitivity	[105]
		Carboxymethyl cellulose (CMC)	An eco-friendly method of repeated freeze–thaw cycles to develop hydrogel composites based on pineapple peel CMC, polyvinyl alcohol, and mesoporous silica SBA-15	[106]
	Ionic interaction	Cellulose microfibrils	Synthesis of cellulose microfibrils hydrogels using the TEMPO-oxidation system with increased storage modulus, compression strength, and surface area	[107]
		Nanocellulose	Development of sugarcane bagasse nanocellulose-based hydrogel as a colorimetric freshness indicator for detecting chicken breast deterioration	[38]
	Hydrogen-bonding interaction	Native cellulose	Facile and low-cost cellulose-based nanocomposite hydrogels with improved mechanical characteristics and adsorption to heavy metal ions utilizing hydroxyapatite (HAP) nanoparticles	[108]
	Complex coacervation	Carboxymethyl cellulose (CMC)	Amaranth protein isolate/CMC complex coacervates develop betanin-containing microcapsules for the creation of edible gelatin films with low light transmission and high antioxidant activity	[109]
	Hydrophobic interaction	Bacterial cellulose (BC)	Synthesis of a sodium alginate-bacterial cellulose-based nanocomposite hydrogel with multi-layered porous surfaces capable of swelling, releasing, and being biocompatible for substrate use	[110]
Chemical crosslinking	Grafting	Hydroxyethyl cellulose (HEC)	Synthesis of self-assembled supermolecular hydrogels based on HEC with potential applications as bacteriostasis materials	[111]
	Crosslinking agents	Carboxymethyl cellulose (CMC)	Crosslinked CMC/gelatin hydrogel films loaded with ZnO nanoparticles using glutaraldehyde as a crosslinking agent with antibacterial and antioxidant characteristics for sustainable food packaging applications	[12]
		Hydroxyethyl cellulose (HEC)	Development of citric acid cross-linked antimicrobial hydrogel films based on HEC and ZnO for food packaging applications	[112]
		Bacterial cellulose (BC)	Crosslinked bacterial cellulose hydrogels with improved mechanical properties and increased water retention capacity employing citric acid and epichlorohydrin as crosslinking agents	[113]
	Enzyme crosslinking	Bacterial cellulose (BC)	Transglutaminase-enzymatic crosslinking of BC/fish collagen composites with increased tensile strength and water vapor permeability	[114]
	Radical polymerization	Carboxymethyl cellulose (CMC)	Fabrication of a superabsorbent hydrogel for water retention and sustained release in advanced agricultural applications	[115]
		Native cellulose	The synthesis of remarkably stretchable and compressible cellulose ionic hydrogels for flexible strain sensors	[116]
	Radiation crosslinking	Hydroxypropyl Methylcellulose (HPMC)	Developed biodegradable HPMC hydrogels with increased strength and swelling qualities using high-energy radiation from electron accelerators.	[117]
		Carboxymethyl cellulose (CMC)	An effective method for synthesizing CMC hydrogels with tailored swelling behavior by varying the radiation dose and the degree of carboxymethylation for targeted applications	[118]

**Table 2:** Key properties, techniques, and significance of CBH characterization in food packaging.

Property	Main Characteristics	Analytical Techniques	Significance	References
Swelling index	Assessment of CBH performance in fluid absorption and swelling behavior	Gravimetric analysis; swelling ratio	Effectiveness of the packaging in preserving the food product by absorbing excess amount of liquid, typically water, and creating a protective barrier around the food product.	[134,136]
Wettability	Examining the degree of interaction between CBHs surfaces and fluids	Contact angle measurement; surface energy measurement; (AFM)	Consideration of wettability of CBHs surface for designing food packaging materials with emphasis on barrier properties and prevention of food product loss or contamination	[137]
Mechanical strength	Investigation of CBHs endurance and performance under specific environmental constraints	Tensile strength; elongation at break; compression strength; rheological characteristics	Preventing physical damage during handling, transportation, and storage by effectively protecting the food product while maintaining the structural integrity and functionality of packaging material under diverse loading conditions	[17,138]
Thermal stability	Thermal analysis under specific temperature conditions	Thermogravimetric analysis (TGA); differential scanning calorimetry (DSC)	Essential for designing food packaging materials to withstand high temperatures during processing, storage, and transportation without compromising the quality and safety of the food product	[12,17]
Physical/morphological characterization	Visualizing the structural features of CBH networks	Scanning electron microscopy (SEM); AFM; field emission scanning electron microscopy (FESEM)	Designing suitable food packaging materials with the optimal porosity, barrier characteristics, and mechanical strength to maintain food quality and safety by visualizing the structural aspects of CBH networks	[134,139,140]
Chemical characterization	Identifying the chemical structures, molecular arrangements, and functional groups of the developed CBHs	Fourier transform infrared (FTIR) spectroscopy; X-ray diffraction (XRD); (NMR) spectroscopy; Raman spectroscopy	Optimizing the performance of CBHs using chemical composition, degree of crystallinity, and molecular orientation of the hydrogels	[134,141,142]

**Table 3:** Recent applications of CBHs for various food products as packaging components.

Packaging Type	Type of Cellulose	Combinations	Characteristics	Applications	References
Hydrogel film	Carboxymethyl cellulose	Polyvinyl alcohol(PVA)/Poly(ethylene imine) (PEI)/Tannic acid (TA)	Hydrogel film with exceptional mechanical strength, self-healing, UV-blocking, strong adhesive strength, antioxidation advantages, and barrier characteristics	Mangoes, strawberries, and cherries	[17]
Hydrogel film	Bacterial cellulose	Polyvinyl pyrrolidone (PVP)/CMC/Guar gum	The remarkable elastic and load-bearing capacity of developed hydrogel films, as well as excellent barrier property and shelf life-enhancing properties of berries up to 15 days	Blueberries	[150]
Hydrogel film	Carboxymethyl cellulose	Gelatin (GEL)/ZnO-NPs	Sustainable hydrogel films with better mechanical properties and good thermal stability with antibacterial activity and antioxidant properties against two food pathogens, *Staphylococcus aureus* and *Escherichia coli*	-	[12]
Hydrogel film	Carboxymethyl cellulose	Cellulose nanofiber/Potassium permanganate	Implementing active hydrogels to enhance the shelf life of bananas by performing as a humidity/ethylene absorbent in the developed food packaging film	Banana	[151]
Hydrogel composite	Bacterial cellulose	Alginate/Cu-NPs	Innovative antimicrobial 3D-printed hydrogel composite that exhibits excellent printability and antibacterial behavior against *Escherichia coli* and *Staphylococcus aureus*	-	[152]
Hydrogel coating	TEMPO-oxidized bagasse cellulose nanofibrils (CNF)	Nisin	Significant reduction in the development of *Listeria monocytogenes* on the external layer of cheese using the developed antimicrobial hydrogel microparticle coating as a paper packaging material	Cheese	[153]
Hydrogel Indicator	Methyl cellulose	Alginate/Bromothymol blue	Hydrogel-based spoilage indicator for minced pork storage: color change upon detection of total volatile basic nitrogen (TVB-N) build-up at 4 °C, demonstrating potential for intelligent packaging applications	Minced pork	[154]
Hydrogel Indicator	TEMPO-oxidized bagasse cellulose nanofibrils (CNF)	Bromothymol blue/Methyl red	Self-supporting CO_2_-sensitive cellulose hydrogel as a colorimetric indicator for food deterioration in intelligent food packaging application	Fresh-cut fruits	[155]
Hydrogel Indicator	Bagasse nanocellulose	Bromothymol blue/Methyl red	Developed hydrogel as a pH-responsive dye carrier used as a colorimetric freshness indicator to track the degradation of chicken breasts as the amount of CO_2_ increased in the headspace	Chicken breasts	[38]
